# Multiple Myeloma DREAM Challenge reveals epigenetic regulator *PHF19* as marker of aggressive disease

**DOI:** 10.1038/s41375-020-0742-z

**Published:** 2020-02-14

**Authors:** Mike J. Mason, Carolina Schinke, Christine L. P. Eng, Fadi Towfic, Fred Gruber, Andrew Dervan, Brian S. White, Aditya Pratapa, Yuanfang Guan, Hongjie Chen, Yi Cui, Bailiang Li, Thomas Yu, Elias Chaibub Neto, Konstantinos Mavrommatis, Maria Ortiz, Valeriy Lyzogubov, Kamlesh Bisht, Hongyue Y. Dai, Frank Schmitz, Erin Flynt, Dan Rozelle, Samuel A. Danziger, Alexander Ratushny, Multiple Myeloma DREAM Consortium, William S. Dalton, Hartmut Goldschmidt, Herve Avet-Loiseau, Mehmet Samur, Boris Hayete, Pieter Sonneveld, Kenneth H. Shain, Nikhil Munshi, Daniel Auclair, Dirk Hose, Gareth Morgan, Matthew Trotter, Douglas Bassett, Jonathan Goke, Brian A. Walker, Anjan Thakurta, Justin Guinney

**Affiliations:** 1 Sage Bionetworks, Seattle, WA USA; 2 Myeloma Center, University of Arkansas for Medical Sciences, Little Rock, AR USA; 3 Computational & Systems Biology, Genome Institute of Singapore, Singapore, Singapore; 4 Celgene Corporation, Summit, NJ USA; 5 GNS Healthcare, Cambridge, MA USA; 6 Celgene, Seattle, WA USA; 7 Department of Computer Science, Virginia Polytechnic Institute and State University, Blacksburg, VA USA; 8 Department of Computational Medicine and Bioinformatics, University of Michigan Medical School, Ann Arbor, MI USA; 9 Shanghai Jiao Tong University, Shanghai, China; 10 Stanford University School of Medicine, Palo Alto, CA USA; 11 Department of Radiation Oncology, Stanford University School of Medicine, Palo Alto, CA USA; 12 Predictive Sciences, Celgene Corporation, Seville, Spain; 13 University of Arkansas for Medical Sciences, Little Rock, AR USA; 14 M2Gen, Tampa, FL USA; 15 Rancho BioSciences, San Diego, CA USA; 16 Department of Malignant Hematology, Moffitt Cancer Center & Research Institute, Tampa, FL USA; 17 Medizinische Klinik V, Universitätsklinikum Heidelberg, Heidelberg, Germany; 18 Nationales Centrum für Tumorerkrankungen, Heidelberg, Germany; 19 Institut Universitaire du Cancer Oncopole, Toulouse, France; 20 Department of Data Sciences, Dana-Farber Cancer Institute, Boston, MA USA; 21 Department of Biostatistics, Harvard TH Chan School of Public Health, Boston, MA USA; 22 Erasmus MC Cancer Institute, Rotterdam, The Netherlands; 23 Department of Malignant Hematology, Moffitt Cancer Center, Tampa, FL USA; 24 Tumor Biology Department, Moffitt Cancer Center, Tampa, FL USA; 25 Department of Medical Oncology, Dana-Farber Cancer Institute, Boston, MA USA; 26 VA Boston Healthcare System, Boston, MA USA; 27 Multiple Myeloma Research Foundation, Norwalk, CT USA; 28 Labor für Myelomforschung, Universitätsklinikum Heidelberg, Heidelberg, Germany; 29 Division of Hematology Oncology, Indiana University, Indianapolis, IN USA

**Keywords:** Risk factors, Myeloma, Myeloma

## Abstract

While the past decade has seen meaningful improvements in clinical outcomes for multiple myeloma patients, a subset of patients does not benefit from current therapeutics for unclear reasons. Many gene expression-based models of risk have been developed, but each model uses a different combination of genes and often involves assaying many genes making them difficult to implement. We organized the Multiple Myeloma DREAM Challenge, a crowdsourced effort to develop models of rapid progression in newly diagnosed myeloma patients and to benchmark these against previously published models. This effort lead to more robust predictors and found that incorporating specific demographic and clinical features improved gene expression-based models of high risk. Furthermore, post-challenge analysis identified a novel expression-based risk marker, *PHF19*, which has recently been found to have an important biological role in multiple myeloma. Lastly, we show that a simple four feature predictor composed of age, ISS, and expression of *PHF19* and *MMSET* performs similarly to more complex models with many more gene expression features included.

## Introduction

Multiple myeloma (MM) is a hematological malignancy of terminally differentiated plasma cells (PCs) that reside within the bone marrow [1]. It arises as a result of complex chromosomal translocations or aneuploidy with ~25,000–30,000 patients diagnosed annually in the United States [2, 3]. The disease’s clinical course depends on a complex interplay of molecular characteristics of the PCs and patient socio-demographic factors. While progress has been made with novel treatments extending the time to disease progression (and overall survival (OS)) for the majority of patients, a subset of 15–20% of newly diagnosed MM patients are characterized by an aggressive disease course with rapid disease progression and poor OS regardless of initial treatment [4–6]. Accurately predicting which newly diagnosed patients are at high risk is critical to designing studies that will lead to a better understanding of myeloma progression and facilitate the discovery of novel therapies that meet the needs of these patients.

To date most MM risk models use patient demographic data, clinical laboratory results and cytogenetic assays to predict clinical outcome. High risk defining cytogenetic alterations typically include deletion of 17p (del(17p)) and gain of 1q as well as t(14;16), t(14;20), and most commonly t(4;14), which leads to juxtaposition of *MMSET* with the immunoglobulin heavy chain locus enhancer, resulting in overexpression of the *MMSET* oncogene [4]. While cytogenetic assays, in particular fluorescence in situ hybridization (FISH), are widely available, their risk prediction is sub-optimal and recently developed classifiers have used gene expression data to more accurately predict risk [7–9]. To investigate possible improvements to models of newly diagnosed myeloma progression, we organized the crowd sourced Multiple Myeloma DREAM Challenge, focusing on predicting high risk, defined as disease progression or death prior to 18 months from diagnosis. This benchmarking effort combined eight datasets, four which provided participants with clinical, cytogenetic, demographic and gene expression data to facilitate model development (*N* = 1624), and four hidden, independent data sets (*N* = 823) for unbiased validation. Over 800 people participated in this Challenge, resulting in the submission of 171 predictive algorithms for objective evaluation. Several models submitted to the Challenge demonstrated improved accuracy over existing state-of-the-art, published models.

Analysis of top-performing methods identified high expression of *PHF19*, a histone methyltransferase, as the gene with the strongest association with myeloma progression, with greater predictive power than the expression level of the known high-risk gene *MMSET*. We developed a four-parameter model using age, ISS, and *PHF19* and *MMSET* expression that performs as well as more complex models having many more gene features. Significantly, we showed that knock down of *PHF19* shifts myeloma cell lines into a less proliferative state. To our knowledge, this is the first DREAM Challenge to both nominate and experimentally validate a candidate biomarker and, as such, demonstrates the biological and clinical impact of crowdsourced efforts.

## Methods

*Datasets:* The Challenge includes five microarray and three RNA-seq expression datasets, annotated with clinical characteristics such as gender, age, International Staging System stage (ISS), and cytogenetics (Table 1) [9–14]. In all datasets, expression assays were performed on CD138+PCs isolated from bone marrow aspirates or blood of newly diagnosed patients. Data were split into training and validation datasets (Table 1).

**Table 1:** Data set descriptions.

					ISS stage			
	Study	EGA/GEO/Clinical trial Id	Median PFS	Data type	1	2	3	*N*
Training datasets	Masaryk [11]	E-MTAB-4032	11.35	Expression array	0.27	0.31	0.42	147
	MAQC-II [10, 14]	GSE24080	25.47	Expression array	0.53	0.26	0.21	559
	MMRF IA9 [31]	NCT01454297	12.59	RNA-seq	0.35	0.37	0.28	636
	HOVON65/GMMG-HD4 [9, 32, 33]	GSE19784	18.3	Expression array	0.43	0.27	0.31	282
	Total training							1624
Validation datasets	MRC-IX [12, 32–35]	GSE15695	*	Expression array	*	*	*	241
	Heidelberg [36, 37]	E-MTAB-372	*	Expression array	*	*	*	215
	Moffitt		*	RNA-seq	*	*	*	74
	DFCI	NCT01191060	*	RNA-seq	*	*	*	293
	Total validation							823

*clinical data withheld per data provider request.

Three institutes provided RNA-sequencing data. The Multiple Myeloma Research Foundation (MMRF) provided an additional training dataset from its publicly available CoMMpass study (release IA9). Collaborating with the Myeloma Genome Project/Dana Farber Cancer Institute (MGP-DFCI) access was provided to data from their clinical trial where patients were randomized into a standard treatment arm and an aggressive treatment arm that included autologous stem cell transplant and high dose therapy [15]. An additional dataset from the Oncology Research Information Exchange Network (ORIEN) was made available through a collaboration with Moffitt Cancer Center and M2Gen (See Supplementary Information for more details on datasets).

### Assessing model performance

To identify top-performing teams we employed two metrics to assess the accuracy of submitted models within a given validation cohort: the integrated area under the curve (iAUC) and balanced accuracy curve (BAC). While the AUC is a widely accepted metric of prediction accuracy, it is sensitive to the specific time threshold used to differentiate high and low patient risk. The myeloma research community has not yet reached a consensus on the time point that best separates patients into risk groups, though there is a general agreement that it lies between 1 and 2 years for progression-free survival (PFS). We, therefore, chose the iAUC “centered” on 18 months as a more robust primary metric. The iAUC range began 6 months prior to 18 months and continued to 6 months past with a sliding PFS threshold moving from12 to 24 months at weekly increments. iAUCs computed in each validation cohort were combined into a weighted average (wiAUC) with each cohort iAUC weighted by the square of the number of high-risk patients in order to ensure larger studies with more high-risk patients did not overwhelm smaller studies while still weighting them more heavily.

Using the wiAUC we computed the Bayes factor, *K*, to identify statistically tied top-performing predictors (see Supplemental Methods). Predictors with *K*_*p*_ *<* *3* are considered tied with the top-scoring model and the weighted BAC (wBAC) was used as a tie-breaking metric in order to determine the final top-performing model, with weighting by the square of the number of high-risk patients in each dataset (see Supplemental text).

### In vitro studies for functional assessment of *PHF19*

Studies to determine the functional importance of PHF19 were performed using standard assays and are described in the Supplemental text. In brief, tetracycline-inducible short hairpin RNA (TRIPZ shRNA) was used to knockdown (KD) *PHF19* expression in two MM cell lines (JJN3 and ARP1). A non-silencing scrambled TRIPZ shRNA was used as a control. *PHF19* KD after doxycycline induction was measured by quantitative real-time polymerase chain reaction (qRT-PCR) and western blotting. Cell viability (Cell Titer Blue, Promega), cell cycle (Vybrant DyeCycle Stain, Thermo Scientific) and apoptosis (Annexin V, Biolegend) were assessed and differences between the *PHF19* KD cells and control group were analyzed.

## Results

### Top Challenge models outperform baseline and published myeloma predictors

**Fig. 1:**
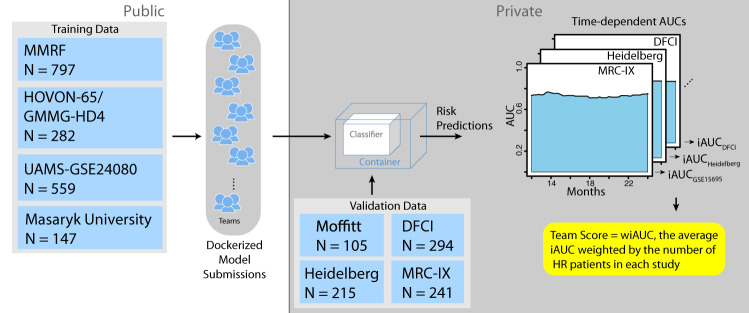
Challenge model submission architecture: training datasets are fully available to Challenge participants (left), while blinded validation datasets are sequestered in the cloud (right). Containerized models are submitted to cloud, ran on training datasets and risk predictions are scored.

To develop and assess prognostic models of high risk in MM, we assembled eight data sets totaling 2447 patients annotated with OS and PFS (Table 1). We asked participants to predict whether a patient was high risk as defined as disease progression or death prior between 12–24 months since diagnosis (see Methods). Participants developed prognostic models using clinical features (e.g., age, sex, ISS, and cytogenetic features) and gene expression utilizing four training datasets. Challenge participants submitted models to be evaluated against four blinded validation cohorts (Fig. 1, Table 1, see Supplemental text). Model predictions were benchmarked against each other and comparator models (Table 2, Supplemental Table 1) using weighted-integrated AUC (wiAUC), with statistical ties resolved using weighted balanced accuracy (wBAC) (see Methods and Supplemental text).

**Table 2:** Comparator modes.

Model	Reference	Features
Baseline	Baseline	Age and/or ISS
UAMS-70	Shaughnessy et al. [7]	Gene expression signature composed of 70 genes
EMC-92	Kuiper et al. [17]	Gene expression signature composed of 92 genes
UAMS-70 extended	This manuscript	UAMS-70 with age and/or ISS
EMC-92 extended	This manuscript	EMC-92 with age and/or ISS
REFS	This manuscript	Gene expression and clinical features

**Fig. 2:**
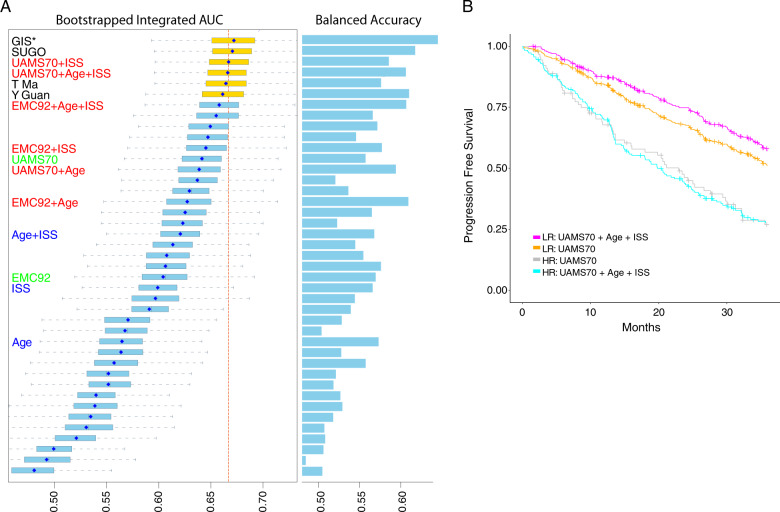
Challenge performance. **a** Box plots show distributions of bootstrapped model performances for each team. Comparator models are shown with text marked in blue for baseline models, green for published models and red for published models extended to include clinical features. The dashed red line indicates the median of the best performing comparator model. Barplots to the right show the tie-breaking metric, wBAC, for each model. Amongst statistically tied models, GIS has the highest wBAC and was declared the top-performer. Asterisk indicates internal collaborator’s comparator model. **b** Kaplan–Meier curve of UAMS-70 comparator model with and without age and ISS added.

Of 42 finalized models submitted to the Challenge, 11 exceeded the performance of the age plus ISS baseline model (Fig. 2, wiAUC = 0.6207). The top-performing predictor, developed by researchers at the Genome Institute of Singapore (GIS), outperformed all Challenge participant models (wiAUC = 0.6721) as well as the published comparator models UAMS-70 (wiAUC = 0.6414) and EMC-92 (wiAUC = 0.6042, Fig. 2).

### Combining clinical features and gene expression features improves myeloma risk prediction accuracy

After the Challenge submission period ended, Challenge organizers and top-performing teams assessed which features had the largest impact on model performance. ISS was the most important model feature in GIS’s top-performing model as measured by the mean decrease in Gini coefficient (see Methods). A DNA repair signature previously associated with poor prognosis [16] was the second most important feature, while age ranked third (Supplemental Fig. 1).

To assess whether age and/or ISS explained the difference in model performance between the top-performing model and published comparator models, we extended the UAMS-70 [7] and EMC-92 [17] models to include age and/or ISS and assessed their performance (Fig. 2; see Supplemental Methods). The addition of these clinical features improved performance of both published models: EMC-92 wiAUC = from 0.6042 vs. EMC-92 + age + ISS wiAUC = 0.658 and UAMS-70 wiAUC = 0.6414 vs. UAMS-70 + ISS wiAUC = 0.6667 as has been suggested previously [13]. Adding ISS to the UAMS-70 model improved its accuracy such that it was tied with the top-performing model (i.e., its Bayes factor *K* < 3; Fig. 2a, b).

### Top-performing Challenge methods identify *PHF19* as a novel myeloma high-risk biomarker

The top-performing model implemented a wisdom of the crowd approach, utilizing clinical features and published myeloma signatures that summarize the expression of gene sets. The second-place “Stanford University Go” (SUGO) model instead included individual genes as features, utilizing a univariate-based feature selection approach to identify genes to include in their model. In each of the four training datasets the SUGO team computed each gene’s effect size, *z*, via the concordance index between OS and the gene’s expression. These effect sizes were combined across training sets using Stouffer’s method [18] without weighting to yield a single meta-*z* per gene. The meta concordance index was calculated using two expression normalization procedures, with one nominating *PHF19* as the most important gene and the second identifying *CDKN3* (See Supplemental Methods).

**Fig. 3:**
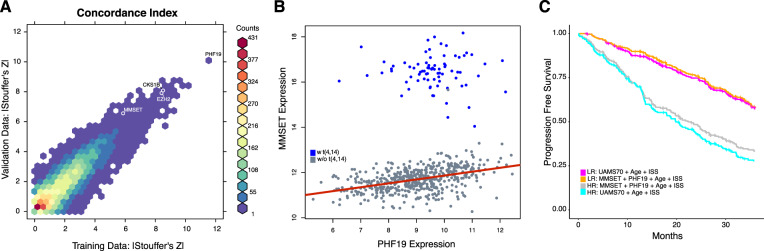
PHF19 compared with other myeloma classifiers and features. **a** Two-dimensional histogram of PFS concordance index-based univariate effect sizes (z) in training and validation cohorts where colors represent the number of genes in a given hexagonal bin. *PHF19* and well-known myeloma genes noted. **b** *PHF19* and *MMSET* expression in relation to t(4;14). **c** A simple four feature model performs as well as UAMS-70 combined with age and ISS.

We replicated SUGO’s feature selection approach in both the training and validation expression datasets. We used the primary end point, PFS, in place of OS when computing each gene’s concordance index given that more patients will progress than die, which results in increased statistical power. We also weighted studies according to their number of high-risk patients when applying Stouffer’s method. This univariate analysis found that previously described myeloma risk genes *MMSET* and *CKS1B* as well as many proliferation genes had large values in the tail of the meta-*z* distribution (Fig. 3a and Supplemental Table 2), validating this approach. The meta-*z* values of these genes and all other genes were surpassed by *PHF19*, the top-ranked gene regardless of normalization procedure (not shown) in both the training and validation datasets (Fig. 3a).

### Incorporating PHF19 and MMSET expression with age and ISS identifies a simple model of high-risk MM

Given that both *PHF19* and *MMSET* are histone modifiers playing a role in H3K36 methylation we checked whether their expression is correlated. As has previously been shown, *MMSET* expression is clearly affected by the t(4;14) translocation with the immunoglobulin enhancer driving high *MMSET* expression, while expression of *PHF19* is not associated with the t(4;14) translocation (Fig. 3b). However, subsetting by t(4;14) reveals a modest linear relationship between *MMSET* and *PHF19* expression in samples lacking the translocation (*r* = 0.423) while there is no such relationship in the t(4;14) positive samples (*r* = −0.067). We also found that higher *PHF19* expression was associated with multiple high-risk genetic factors such as IGH translocation groups, non-hyperdiploidy, TP53 mutations and the overall number of drivers per sample (*p* < 0.001, Supplemental Fig. 2).

Given the impact of *PHF19* on model performance and *MMSET*’s status as a reported myeloma risk marker, we checked to see if a Cox proportional-hazards model composed of age, ISS, *PHF19* and *MMSET* could perform as well as a similar model using the features of the top-performing extended comparator model (UAMS-70 plus age plus ISS). We constructed Cox proportional-hazards models of the two feature sets and found that the four-parameter model (wiAUC = 0.693, Supplemental Table 3) out-performed the UAMS-70-based model in the validation cohort (wiAUC = 0.667; Fig. 3c) placing it on par with the winning algorithm.

### Knockdown of *PHF19* leads to decreased proliferation through cell cycle arrest in multiple myeloma cell lines

**Fig. 4:**
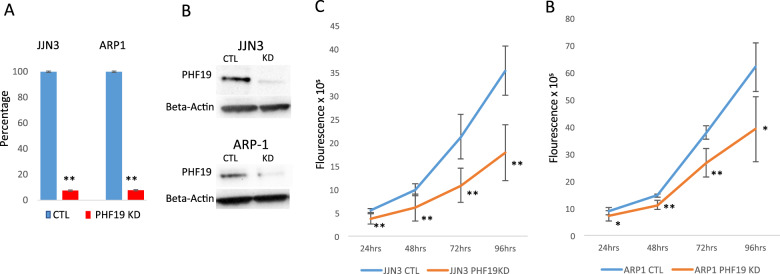
PHF19 knockdown leads to decreased cell: knockdown of PHF19 was performed in the JJN3 and ARP1 MM cell lines using inducible shRNA. **a** PHF19 knockdown, relative to scrambled shRNA control, was confirmed using qRT-PCR and **b** western blotting. **c**, **d** Cell proliferation was significantly decreased in MM cells with PHF19 knockdown compared with scrambled control for JJN3 and ARP1 cell lines.

To determine whether *PHF19* is functionally important for the malignant growth of MM cells, we used lenti viral-expressed shRNA directed against *PHF19*. We transduced JJN3 and ARP1 MM cell lines with a shRNA targeting *PHF19* or a scrambled control shRNA and selected out transduced cells. shRNA induced cells showed KD of >90% *PHF19* RNA and protein relative to the control after 72 h and 168 h for the JJN3 and ARP1 cell lines, respectively (Fig. 4a, b). KD of *PHF19* led to significant inhibition of proliferation in the JJN3 and ARP1 MM cell lines compared with scrambled shRNA control (Fig. 4c, d) confirming the recent finding of PHF19’s effect on proliferation in MM cell lines [19]. To identify the mechanism of growth inhibition, we performed cell cycle analysis and observed a significant arrest of MM cells in the G0/G1 stage with *PHF19* KD compared with the scrambled control shRNA (Supplemental Fig. 3a). This was seen consistently in both cell lines examined (Supplemental Fig. 3b). We further investigated the effect of *PHF19* KD on apoptosis and necrosis, but did not find significant differences at the examined time points (Supplemental Fig. 3c–e). These results demonstrate that *PHF19* is functionally relevant in MM and that reduction of *PHF19* leads to a decrease in cell proliferation via cell cycle arrest.

## Discussion

In the course of the crowd sourced Multiple Myeloma DREAM Challenge we benchmarked 171 prediction models and found that the accuracy of gene expression-based models benefited from the addition of clinical data, specifically: age and ISS improved AUC-based metrics by ~6%. As such, expression-based patient stratification efforts should incorporate age and ISS.

In addition, we show for the first time that expression of *PHF19* is a stronger predictor of MM progression than the expression level of the high-risk marker *MMSET*, which is particularly overexpressed in patients with the high-risk translocation t(4;14). This strong association was likely missed in earlier studies given that *PHF19* expression is not associated with any specific cytogenetic feature while several therapeutic advances over the last 20 years have made it difficult to model outcome across multiple studies from different periods. Furthermore, *PHF19* has not been found to be significantly mutated in sequencing-based studies [20, 21], suggesting that its overexpression is not directly related to genomic alterations within the *PHF19* gene. We also show that a simple four feature predictor composed of age, ISS, and expression of *PHF19* and *MMSET* performs similarly to more complex models that are based on RNA-seq and gene expression analysis, which have been difficult to implement into the clinical setting (Supplemental Fig. 4). In contrast, the quantitative expression of *PHF19* and *MMSET* could be easily measured by readily available methods such as real-time PCR, suggesting that the simplicity of the current model could be more easily adopted in a general clinical setting.

Apart from its prognostic value, we show that *PHF19* has functional importance in MM. KD of *PHF19* led to a significant reduction of growth and cell cycle arrest ex vivo, suggesting that *PHF19* may play a role in transitioning cells into a highly proliferative state in MM. *PHF19* has been shown to be a major modulator of histone methylation thereby regulating transcriptional chromatin activity [22] with a known role in B-cell differentiation into plasma cells within the germinal center [23, 24]. *PHF19* directly recruits the polycomb repressive complex 2 (PRC2) via binding to H3K36me3 and leads to activation of enhancer of zeste homolog 1 and 2 (EZH1/EZH2) as enzymatic subunits of PRC2, thereby resulting in tri-methylation of H3K27 [25, 26]. This process has been shown to enforce gene repression and is known to promote tumor growth in a variety of cancers [27]. While *MMSET* has also been shown to regulate histone methylation, its role as an epigenetic modulator is less well understood. Some reports have suggested that *MMSET* leads to transcriptional repression through generation of H4K20me [28], H3K27me3 [29] or H3K36me3 [29], while other studies show that *MMSET* enhances transcription through generation of H4K20me2 [30] and H3K36me2 [29]. In contrast to *MMSET*, *PHF19* expression is present in all MM subgroups and is preferentially overexpressed in high-risk MM. These results are indicative of a strong correlation between increased histone methylation, in particular H3k27 trimethylation, and disease aggressiveness. Further work will be necessary to elucidate the mechanisms of *PHF19* in MM biology and any interplay with *MMSET*.

## Supplementary information

Supplemental Text

Supplemental Figure 1

Supplemental Figure 2

Supplemental Figure 3

Supplemental Figure 4
