# Supplementary figures and images for: Multiple Myeloma DREAM Challenge reveals epigenetic regulator PHF19 as marker of aggressive disease
Source: Leukemia. 2020 Feb 14;34(7):1866–74. doi: 10.1038/s41375-020-0742-z (PMC7326699; doi:10.1038/s41375-020-0742-z)

A

## GIS Feature Importance

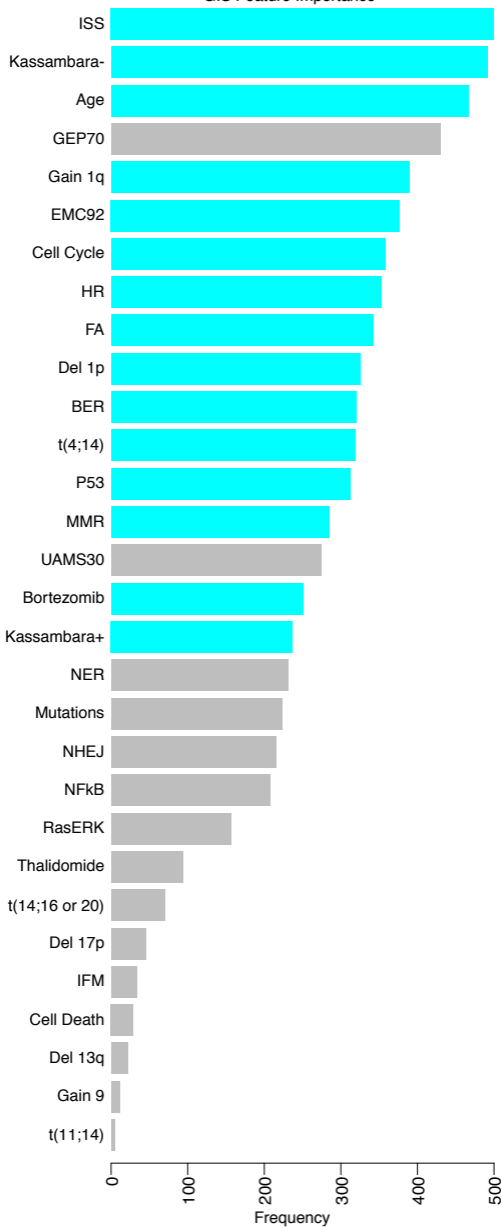

Supplement: Supplementary file 2 — Supplemental Figure 1 [file 41375_2020_742_MOESM2_ESM.pdf]

# Cell Cycle

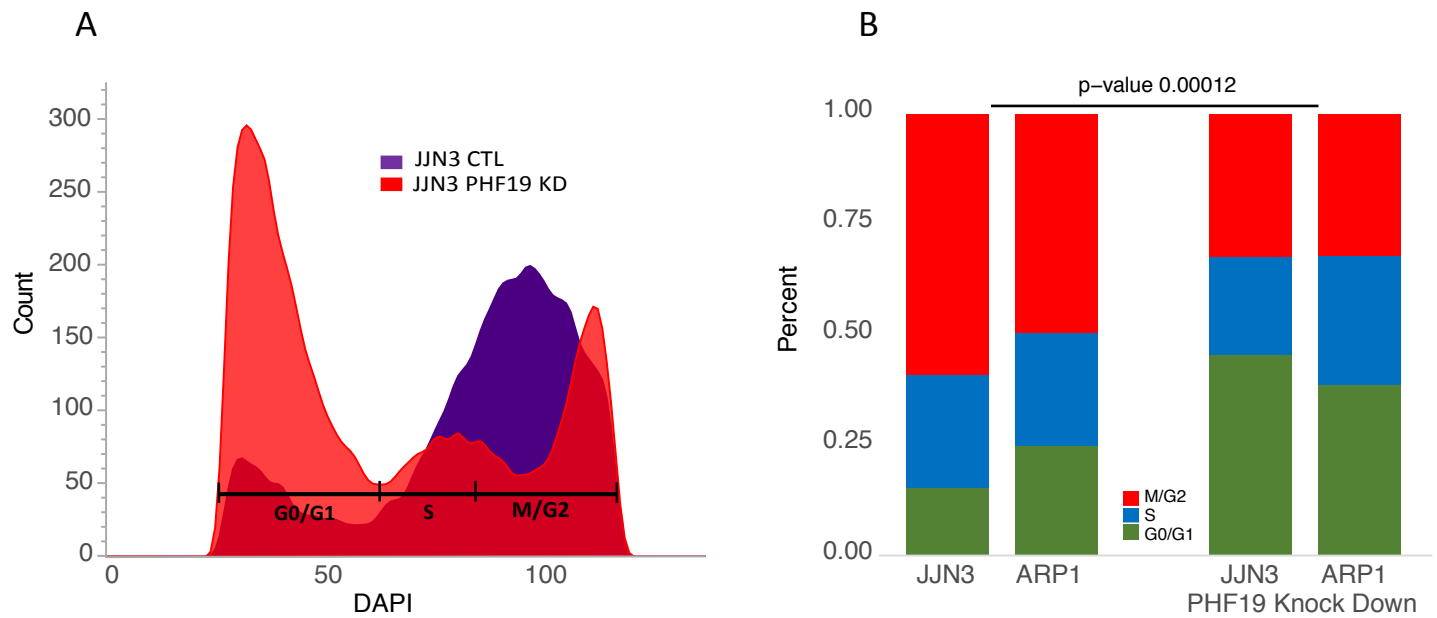

# Apoptosis

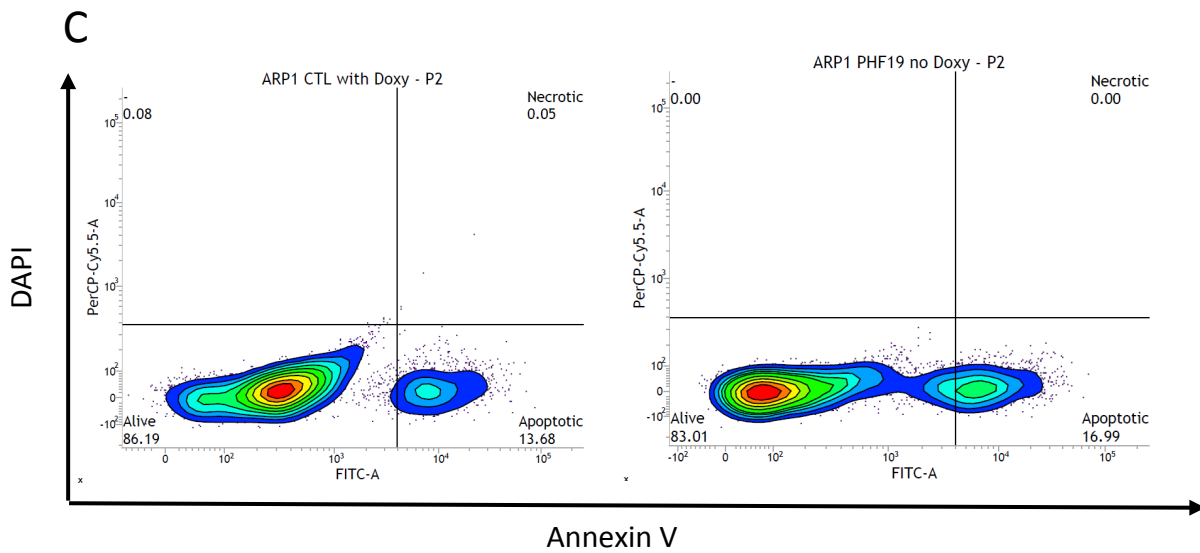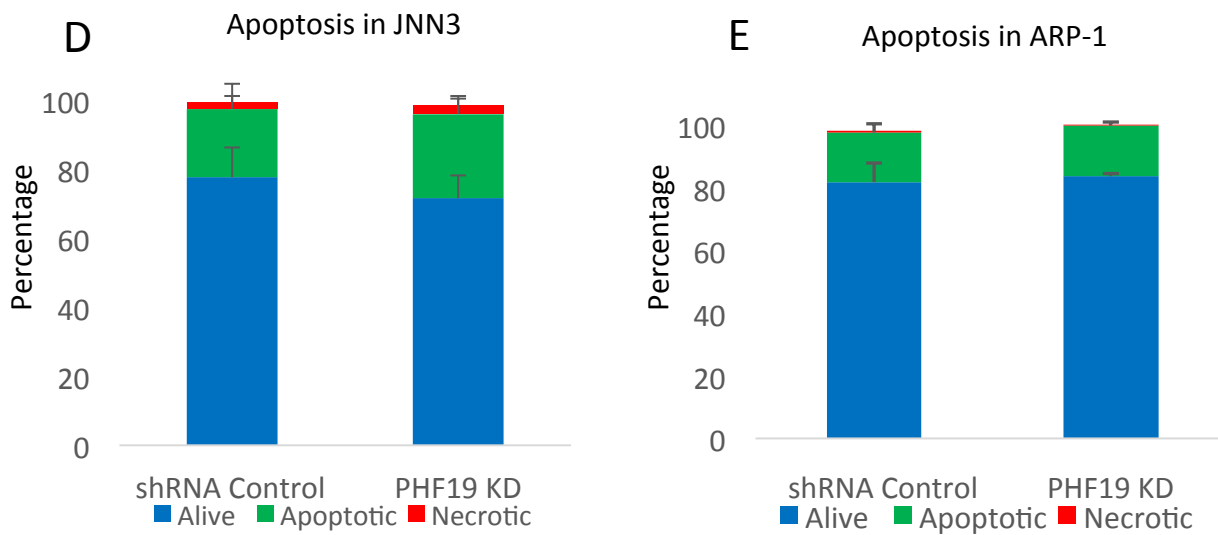

Supplemental Figure 3

Supplement: Supplementary file 4 — Supplemental Figure 3 [file 41375_2020_742_MOESM4_ESM.pdf]

**DFCI Cor = 0.77**

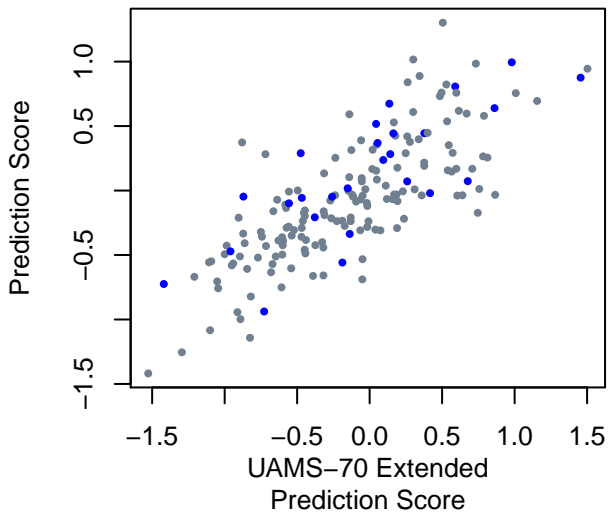

**Heidelberg Cor = 0.88**

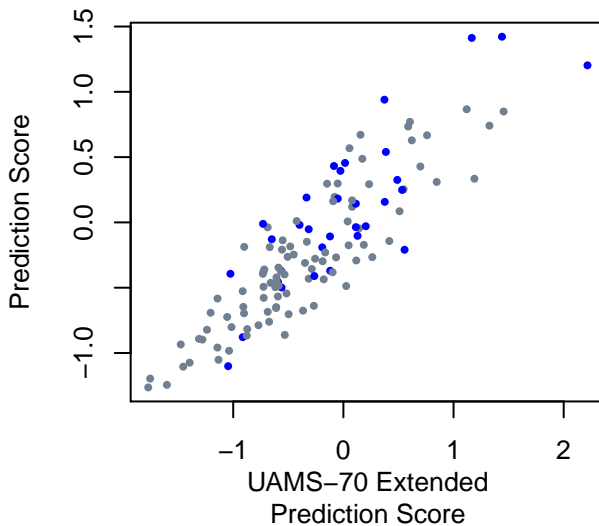

**M2Gen Cor = 0.8**

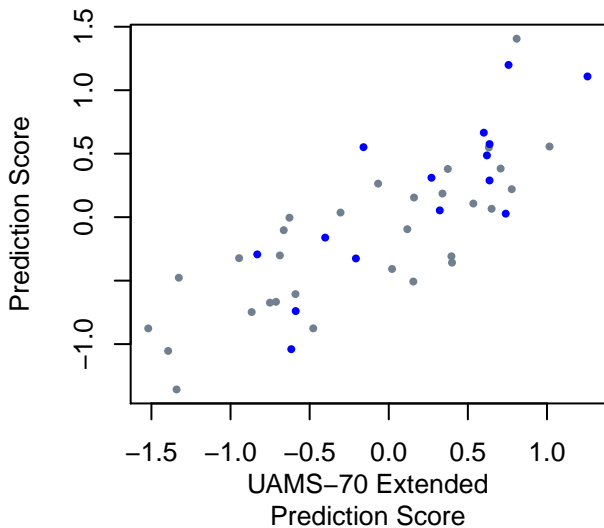

**MRC-IX Cor = 0.88**

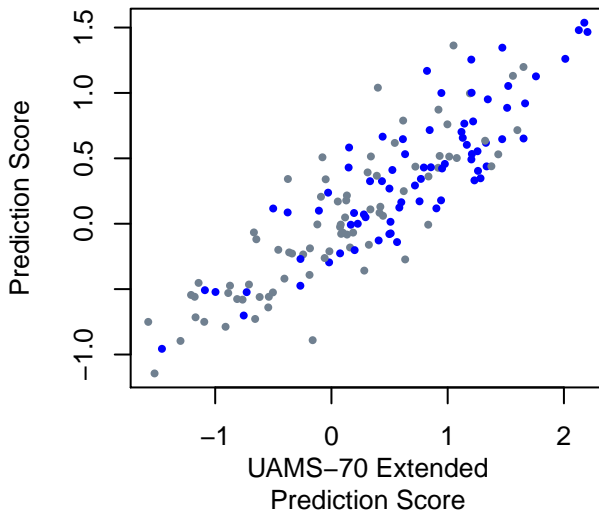

Supplement: Supplementary file 5 — Supplemental Figure 4 [file 41375_2020_742_MOESM5_ESM.pdf]
