# Supplementary material for: Multiple Myeloma DREAM Challenge reveals epigenetic regulator PHF19 as marker of aggressive disease
Source: Leukemia. 2020 Feb 14;34(7):1866–74. doi: 10.1038/s41375-020-0742-z (PMC7326699; doi:10.1038/s41375-020-0742-z)
Supplement: Supplementary file 3 — Supplemental Figure 2 [file 41375_2020_742_MOESM3_ESM.pdf]

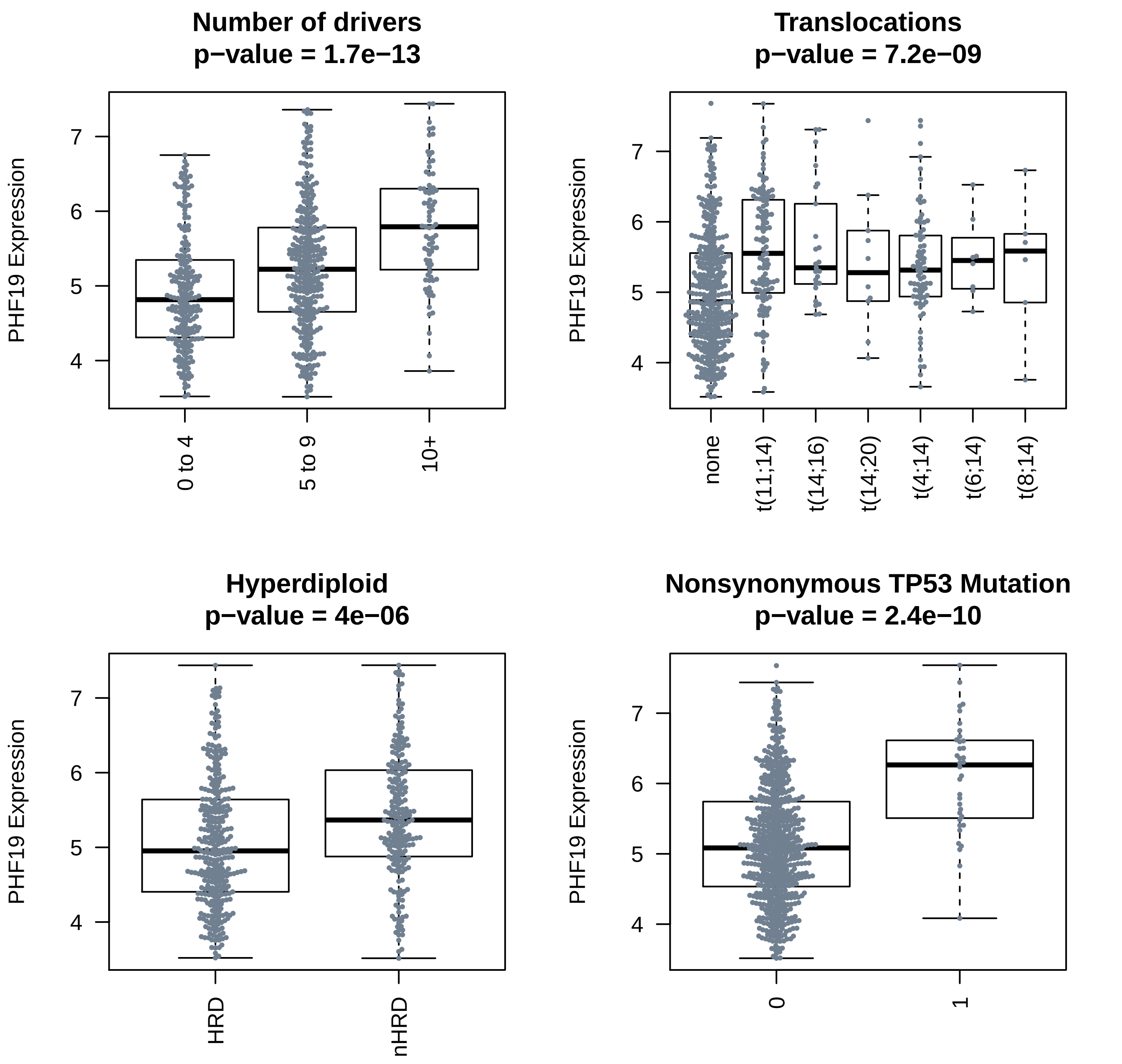
Number of drivers
p−value = 1.7e−13
PHF19 Expression
4
5
6
7
0 to 4
5 to 9
10+
Translocations
p−value = 7.2e−09
PHF19 Expression
4
5
6
7
none
t(11;14)
t(14;16)
t(14;20)
t(4;14)
t(6;14)
t(8;14)
Hyperdiploid
p−value = 4e−06
PHF19 Expression
4
5
6
7
HRD
nHRD
Nonsynonymous TP53 Mutation
p−value = 2.4e−10
PHF19 Expression
4
5
6
7
0
1
